# Role of Cyclin-Dependent Kinase 4/6 in Metastatic Breast Cancer: Real-World Data From a Tertiary Care Institute in Eastern India

**DOI:** 10.7759/cureus.52172

**Published:** 2024-01-12

**Authors:** Saroj Kumar Das Majumdar, Sandip Kumar Barik, Ashutosh Pattanaik, Deepak Kumar Das, Dillip Kumar Parida

**Affiliations:** 1 Radiation Oncology, All India Institute of Medical Sciences Bhubaneswar, Bhubaneswar, IND; 2 Radiation Oncology, Apollo Hospitals Bhubaneswar, Bhubaneswar, IND

**Keywords:** abemaciclib, ribociclib, palbociclib, real world evidence metastatic breast cancer, cyclin dependent kinase 4/6 inhibitors, metastatic hormone positive breast cancer

## Abstract

Introduction

CDK4/6 inhibitors currently approved for patients with hormone-receptor-positive (HR+)/human epidermal growth factor receptor 2-negative (HER2-) metastatic breast cancer include palbociclib, ribociclib, and abemaciclib. This study aims to report on the treatment outcomes and real-world data regarding the use of CDK4/6 inhibitors in the treatment of ER+/HER2- metastatic breast cancer at a tertiary care institute in Eastern India.

Materials and methods

The present study is a retrospective analysis of data from patients with metastatic HR+/HER2- breast cancer who were treated with CDK4/6 inhibitors at a tertiary care institute in Eastern India between 2015 and 2022. The data were collected from online records in the departmental files and analyzed for the primary baseline characteristics of the patients, tumors, and response rates, including partial response (PR), complete response (CR), progressive disease (PD), and stable disease (SD), as defined by the Response Evaluation Criteria in Solid Tumors (RECIST) criteria version 1.1. The treatment administered, progression-free survival (PFS), and toxicity were also evaluated.

Results

From 2015 to 2022, 24 eligible patients were treated with CDK4/6 inhibitors for metastatic HR+/HER2- breast cancer. The average duration of follow-up was 25 months. Out of the 24 patients, 15 (62.5%) were taking Tab. ribociclib, six (25%) were taking Tab. palbociclib, and three (12.5%) were taking Tab. abemaciclib. CDK4/6 was used as a first-line therapy for 16 patients, while eight patients received it as a second-line treatment. Out of the total number of patients, six (25%) had stable disease, 13 (54.2%) had a partial response, and four (16.7%) had progressive disease. In total, of the eligible patients, five (20.8%) had grade I neutropenia, seven (29.2%) had grade II neutropenia, and four (16.7%) had grade III neutropenia. At five years, the PFS rate estimated by the Kaplan-Meier method was 50% (95% CI: 47.89-69.31).

Conclusion

Ribociclib and palbociclib have improved PFS in patients with metastatic HR+/HER2- breast cancer. Both drugs have well-tolerated toxicity, allowing patients to continue taking them for an extended period of time. CDK4/6 inhibitors have a higher response rate than the other agents.

## Introduction

The treatment of metastatic hormone-receptor-positive (HR+) disease focuses on targeting the estrogen receptor signaling pathway with hormonal therapy or chemotherapy [1]. The development of cyclin-dependent kinase 4/6 (CDK4/6) inhibitors for the treatment of hormone receptor-positive advanced breast cancer was based on preclinical studies that identified a dependence of HR+ breast cancer on CDK4 and CDK6 signaling as well as a synergistic effect from targeting the ER, cyclin-D-CDK4/6-Rb pathway [2].

In India, breast cancer accounted for 13.5% of diagnosed malignancies and 10.6% of deaths due to malignancies in 2020, making it the most prevalent cancer in terms of both incidence and mortality [3]. According to global cancer statistics, 50-75% of Asian women are diagnosed with HR+/human epidermal growth factor receptor 2-negative (HER2-) breast cancer [4,5]. The median age of Asians at the time of breast cancer diagnosis, which is between 45 and 50 years, is lower than that of their Western counterparts, who are typically diagnosed between 55 and 60 years of age [6,7]. The rate of breast cancer in premenopausal women is higher in Asian populations compared to non-Asian populations [8,9]. Response rates and adverse reactions may differ between Asian and non-Asian patients due to genetic variations, pharmacogenomics, and drug metabolism [10].

The PALOMA-2 trial confirmed that the combination of palbociclib and letrozole leads to significantly longer progression-free survival than letrozole alone in postmenopausal women with advanced breast cancer, i.e., HR+ and HER2-. The study also provided further evidence of the efficacy and safety of inhibiting CDK4 and CDK6 as a first-line treatment [3]. The treatment paradigm for HR+ and HER2- breast cancer has undergone a significant shift with the discovery of CDK4/6 inhibitors. These inhibitors are now considered the first- or second-line treatment for these patients [11-13].

CDK4/6 inhibitors currently approved for patients with HR+/HER2- metastatic breast cancer include palbociclib, ribociclib, and abemaciclib. In India as well, oncologists have started to adopt these treatment protocols. However, many people faced difficulties due to the high cost of the therapy, which imposed a significant financial burden on patients. Therefore, this study aims to report on the treatment outcomes and real-world data regarding the use of CDK4/6 inhibitors in the treatment of HR+/HER2- metastatic breast cancer at a tertiary care institute in Eastern India.

## Materials and methods

Study design

The present study is a retrospective data analysis of patients with metastatic HR+/HER2- breast cancer who were treated with CDK4/6 inhibitors at a tertiary care institute in Eastern India between 2015 and 2022.

Study methodology

The data were collected from online records in the departmental files and analyzed for primary baseline characteristics of the patient and tumor. All patients with metastatic HR+ and HER2- breast cancer who were either upfront or on progression treated with a CDK4/6 inhibitor and hormonal therapy were included in the study. If the patient was pre or peri-menopausal, ovarian ablation was offered. Patients treated with chemotherapy in previous settings and subsequently having progressive disease were treated with CDK4/6 as second-line therapy. The primary objective of the study was response rates, including partial response (PR), complete response (CR), progressive disease (PD), and stable disease (SD), as defined by the RECIST criteria version 1.1. The treatment administered, progression-free survival (PFS), and toxicity were also evaluated. PFS is the time from the start date of CDK4/6 therapy to the first documented clinical and radiological progression or death due to any cause. Toxicity was assessed according to Common Terminology Criteria for Adverse Events (CTCAE) version 4. The study was exempt from review by the Institute Ethics Committee due to its retrospective nature. This exemption was granted in a letter dated 19/11/2022 with the reference number T/IM-NF/Radioth/22/92.

Statistical analysis

Baseline patient and disease-specific characteristics were compared using the chi-square test. PFS was calculated using the Kaplan-Meier method, and survival comparisons were conducted using the log-rank test. Univariate analyses (using log-rank tests) and multivariate analyses (using the Cox proportional hazards model) were used to assess the impact of known prognostic variables on PFS. A P-value of <0.05 was considered statistically significant. All analyses were performed using SPSS version 25.0 (IBM Corp., Armonk, NY, USA).

## Results

Patient characteristics

From 2015 to 2022, 24 eligible patients were treated with CDK4/6 inhibitors for metastatic estrogen receptor-positive (ER+)/HER2- breast cancer. All of the patients were female. The median age was 51 years, ranging from 39 to 79 years. The mean follow-up duration was 25 months, with a median of 20 months and a standard deviation of 21.499. Eighteen (75%) patients had bone metastases, and 13 patients had lung metastases, of whom seven had both lung and bone metastases. Out of the total number of patients, four had liver metastases. Among these, three had both liver and bone metastases while one had liver and lung metastases. Eight patients were premenopausal, and 16 were postmenopausal. Out of eight premenopausal patients, three underwent a surgical oophorectomy. Eight patients had prior chemotherapy with a combination of taxanes, adriamycin, and cyclophosphamide. Six patients were taking tamoxifen, 11 were on letrozole, and two were on anastrozole. Five patients were on fulvestrant as their first line of treatment, and four patients were switched from aromatase inhibitors (AI). One patient was taking exemestane (Table 1).

**Table 1:** Depicting the patient's characteristics

Factor	Patients (n=24) (100%)
Age, Median (min-max)	51.54 (39 yrs to 79 yrs)
Laterality	
Bilateral	2 (8.3)
Left	9 (37.5)
Right	13 (54.2)
Co-morbidities	
Diabetes	1 (4.2)
Diabetes with hypertension	1 (4.2)
Hypertension	6 (25)
Hypertension with hyperthyroidism	1 (4.2)
No co-morbidity	15 (62.5)
Tumor stage	
T0	3 (12.5%)
T1	3 (12.5%)
T2	8 (33.3%)
T3	1 (4.2%)
T4	9 (37.5%)
Nodal staging	
N0	7 (29.2%)
N1	12 (50%)
N2	1 (4.2%)
N3	4 (16.7%)
Metastases	
Patients with metastases	24 (100%)
Bone metastases	
No bony metastases	6 (25%)
With bony metastases	18 (75%)
Oligometases	
Nil	24 (100%)
Estrogen receptors	
Present (ER +ve)	24 (100%)
Progesterone receptors	
Absent	3 (12.5%)
Present	21 (87.5%)
HER2 receptors	
Absent	24 (100%)
Systemic chemotherapy	
Not administered	16 (66.7%)
Administered	8 (33.3%)
Oophorectomy	
No	19 (79.2%)
Yes	5 (20.8%)
Previous endocrine therapy	
Not received	13 (54.2%)
Received	11 (45.8%)
Fulvestrant	
Not administered	14 (58.3%)
Administered	10 (41.7%)
Aromatase inhibitors	
Not administered	3 (12.5%)
Administered	21 (87.5%)

CDK4/6 inhibitors

Out of the 24 patients, 15 (62.5%) were taking Tab. ribociclib, six (25%) were taking Tab. palbociclib, and three (12.5%) were taking Tab. abemaciclib. CDK4/6 was used as a first-line therapy for 16 patients while eight patients received it as a second-line treatment. The mean duration of palbociclib continuation was 20.33 months, whereas for ribociclib, it was 15.6 months, and for abemaciclib, it was only two months. Six patients received the ribociclib and fulvestrant combination while seven received ribociclib and AI. Among the patients who received palbociclib, four received it in combination with fulvestrant, and two received it with AI. In addition, three patients who received abemaciclib were also treated with an AI (Table 1).

Response rates

Among all the patients who received CDK4/6 inhibitors, the overall response rate (ORR) was 58.4%. One (4.2%) achieved a complete response with ribociclib, six (25%) had stable disease, 13 (54.2%) had a partial response, and four (16.7%) had progressive disease (Table 2).

**Table 2:** Response rates with CDK4/6 inhibitors in metastatic breast cancer

RESPONSE RATES	Patients (n=24) (100%)
Complete metabolic response	1 (4.2%)
Progressive disease	4 (16.7%)
Partial response	13 (54.2%)
Stable disease	6 (25%)

Toxicity

Grade of neutropenia: Five (20.8%) patients had grade I neutropenia, with four patients receiving ribociclib and one patient receiving palbociclib; seven (29.2%) patients had grade II neutropenia, with six patients receiving ribociclib and one patient receiving palbociclib; and four (16.7%) patients had grade III neutropenia, with three patients receiving palbociclib and one patient receiving ribociclib.

Additional toxicity results: Grade I anemia was found in 13 (54.2%) patients; generalized rashes were found in two (8.3%) patients; gastrointestinal toxicity was found in nine (37.5%) patients; and grade I hepatotoxicity was found in seven (29.2%) patients (Table 3).

**Table 3:** The various toxicities found in the study

Toxicities	Patients (n=24) (100%)
Anemia	13 (54.2%)
Neutropenia	
Present	16 (66.7%)
Grade of neutropenia	
Gr-1	5 (20.8%)
Gr-2	7 (29.2%)
Gr-3	4 (16.7%)
Fatigue	
Yes	19 (79.2%)
Skin rashes	
Present	2 (8.3%)
Gastrointestinal toxicity	
Present	9 (37.5%)
Grade	
1	5 (20.8%)
2	4 (12.5%)
QT prolongation	
Present	1 (4.16%)
Hepatotoxicity	
1	7 (29.2%)
Grade	
1	7 (29.2%)
Thrombosis	
Present	2 (8.3%)

Univariate analysis

The univariate Cox regression analysis is shown in Table 4. No variable was found to be statistically significant.

**Table 4:** Univariate analysis of different variables

Factor	Survival Percentage,(85% CI)	P-value
Age		0.5628
<50 yrs	87.5 (38.7-0.9814)	
>50 yrs	100	
Bilateral		0.1264
Left	100	
Right	90 (47.3-0.9853)	
Comorbidity		
Diabetes mellitus	100	0.9825
Diabetes mellitus and hypertension	100	
Hypertension	100	
Hypertension and hyperthyroidism	100	
NO	90 (47.3-0.9853)	
T Stage		0.9028
T0	100	
T1	100	
T2	100	
T3	100	
T4	66.67 (5.41-0.9452)	
N Stage		0.2582
N0	100	
N1	85.71 (33.41-0.9786)	
N2	100	
N3	100	
M Stage		
M1	92.86 (59.08-0.9896)	
Bone		0.4014
Yes	100	
No	90.91 (50.81-0.9867)	
Progesterone receptors		0.8193
No	100	
Yes	91.67 (53.9-0.9878)	
Systemic chemotherapy		0.1942
No	100	
Yes	83.33 (27.31-0.9747)	
Oophorectomy		0.2270
No	91.67 (53.9-0.9878)	
Yes	100	
Previous endocrine therapy		
No	80 (20.38-0.9692)	0.4453
Yes	100	
Fulvestrant		0.0693
No	100	
Yes	87.5 (38.7-0.9814)	
Aromatase inhibitors		0.2814
No	100	
Yes	90.91 (50.81-0.9867)	
Anemia		0.6081
No	100	
Yes	88.89 (43.3-0.9836)	
Neutropenia		0.5038
No	100	
Yes	90.91 (50.81-0.9867)	
Grade of neutropenia		0.5078
0	100	
1	100	
2	80 (20.38-0.9692)	
3	100	
Fatigue		0.6750
No	100	
Yes	90 (47.3-0.9853)	
Rash		0.2777
No	100	
Yes	50 (0.6-0.9104)	
Gastrointestinal toxicity		0.0805
No	100	
Yes	83.33 (27.31-0.9747)	
GRADE		0.7439
0	100	
1	100	
2	66.67 (5.41-0.9452)	
Hepatotoxicity		0.4190
No	100	
Yes	92.86 (59.08-0.9896)	

Progression-free survival

**Figure 1:**
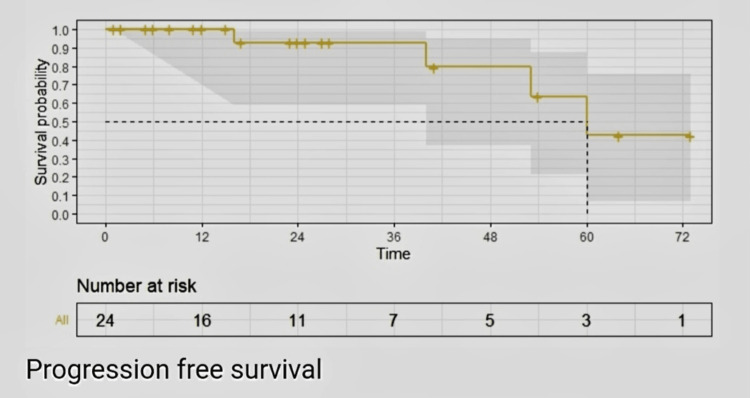
Progression-free survival by Kaplan and Meier estimates

At five years, PFS by the Kaplan and Meier method was estimated to be 60%, 95% CI (47.89-69.31). All patients were alive by this study's analysis; hence, the overall survival rate was not reported (Figure 1).

## Discussion

Among women with hormone receptor-positive breast cancer, combinations of AIs such as letrozole with CDK4/6 inhibitors (palbociclib, ribociclib, and abemaciclib) have demonstrated improved PFS relative to AIs alone [14]. The US Food and Drug Administration (FDA) has approved these drugs as a first or second-line treatment. Furthermore, a meta-analysis of nine randomized trials involving over 5000 postmenopausal patients revealed that the addition of CDK 4/6 inhibitors to ET resulted in improved OS (hazard ratio [HR] 1.33, 95% CI: 1.19-1.48). Nevertheless, it also increases the risk of neutropenia, leukopenia, and diarrhea [15]. The response rates of the three CDK4/6 inhibitors have not been directly compared in any clinical trials. However, a meta-analysis found no statistically significant differences in PFS among the three CDK 4/6 inhibitors when combined with an AI [16]. Palbociclib and ribociclib have been found to have higher rates of neutropenia than abemaciclib. On the other hand, abemaciclib is more commonly associated with causing diarrhea. Ribociclib has demonstrated an OS benefit when added to an AI or AI plus ovarian function suppression in the MONALEESA-2 trial (64 months versus 51 months, HR 0.76, 95% CI: 0.63-0.93) [17] and the MONALEESA-7 trial (58 months versus 48 months, HR 0.76, 95% CI: 0.61-0.96) [18]. Although palbociclib did not demonstrate an OS benefit in the PALOMA-2 trial (54 months versus 51 months, HR 0.96, 95% CI: 0.78-1.2) [19], a combined analysis of PALOMA-1 and PALOMA-2 showed an OS benefit in the group that had disease-free intervals of over 12 months [20]. The MONARCH-3 trial showed a trend towards longer OS by adding abemaciclib to an AI (67 months versus 55 months, HR 0.75, 95% CI: 0.58-0.97) [21]. However, the results for overall survival are still pending.

The PALOMA-3 study included both premenopausal and postmenopausal Asians who were administered either palbociclib plus fulvestrant (n = 71) or placebo plus fulvestrant (n = 31). Palbociclib in combination with fulvestrant has been shown to improve progression-free survival (PFS) compared to treatment with fulvestrant alone. The median PFS was not reached with palbociclib plus fulvestrant (95% CI, 9.2 months to not reached). In contrast, it was only 5.8 months with placebo plus fulvestrant (95% CI, 3.5 to 9.2 months; hazard ratio 0.485; 95% CI: 0.270-0.869; P = .0065). The most frequent grade 3 or 4 adverse events, regardless of cause, in the palbociclib group were neutropenia (92%) and leukopenia (29%) [22].

Fulvestrant, in combination with a CDK4/6 inhibitor, may be a viable alternative for individuals who cannot tolerate AI-based therapy. In a randomized trial of 486 patients with previously untreated, hHR+/HER2- advanced breast cancer, fulvestrant plus palbociclib was compared to letrozole plus palbociclib [23]. The median PFS was 27.9 months for the fulvestrant-palbociclib combination, compared to 32.8 months for letrozole-palbociclib. However, this difference was not statistically significant. In a pooled analysis conducted by the FDA, two trials were evaluated to determine the effectiveness of combining CDK 4/6 inhibitors or placebo with fulvestrant in 396 patients. The estimated HR for overall survival was 0.74 (95% CI: 0.52-1.07), indicating a favorable outcome with the addition of CDK 4/6 inhibitors [24].

In the present study, the majority of our experience is with ribociclib (62.5%), followed by palbociclib (25%), and abemaciclib (12.5%). The majority of patients received CDK4/6 inhibitors as their first-line therapy. The mean duration of palbociclib continuation was 20.33 months, whereas for ribociclib, it was 15.6 months, and for abemaciclib, it was only two months. Response rates were as follows: six (25%) patients had stable disease, 13 (54.2%) patients had a partial response, and four (16.7%) patients had progressive disease. Out of the total number of patients, five (20.8%) had grade I neutropenia, seven (29.2%) had grade II neutropenia, and four (16.7%) had grade III neutropenia. At five years, the PFS was estimated to be 50% (95% CI: 47.89-69.31) using the Kaplan-Meier method.

In a study conducted in eastern India with 144 patients who were treated with CDK4/6 inhibitors, the overall response rate was observed in 61% of patients, and the clinical benefit rate was observed in 79%. At a median follow-up of 20.2 (95% CI: 13.5-28.3) months, the median PFS of the study population was 16.5 (95% CI: 11.6-25.5) months, and the median OS of the study population was 29.7 (95% CI: 21.7-44.6) months [25].

In another study conducted in India, an analysis of 122 patients revealed that the median PFS was 18 months (ranging from 4-36 months). Twenty-four (20%) patients achieved a complete response, 69 (57.5%) patients attained a partial response, 18 (15%) patients had stable disease, and nine (7.5%) experienced disease progression [26].

Rath et al. conducted a retrospective study of 101 patients who received either palbociclib or ribociclib [27]. In the first-line treatment, with a median follow-up of 21.7 (0.5-41.9) months, the median PFS and OS were 21.1 (95% CI: 16.36-not estimable) months and not reached, respectively. In the second- or later-line setting, with a median follow-up of 17.2 (0.5-43.7) months, the median PFS and OS were 5.98 (95% CI: 4.96-7.89) months and 20.2 (95% CI: 14.1-not estimable) months, respectively. Grades 3-4 neutropenia and febrile neutropenia were observed in 45 (45.0%) and 9 (9.0%) patients, respectively.

Shortcomings of the study

The study had a small sample size due to cost constraints associated with the use of CDK4/6 therapy. The high cost of the treatment made it difficult to secure sufficient finances for a larger sample size. The government schemes covered the CDK4/6 cost only in 2022. The coronavirus disease 2019 (COVID-19) pandemic has had a significant impact on recruitment from 2019 to 2021. Many patients were either forced or preferred to stay at home [28]. In the year 2023, palbociclib costs will have been drastically reduced in India, and there will now be many generic forms of the drug available. Hopefully, many more patients can benefit from treatment with CDK4/6 inhibitors.

## Conclusions

CDK4/6 inhibitors have improved the PFS in patients with metastatic ER+/HER2- breast cancer. Both drugs have well-tolerated toxicity, allowing patients to continue taking them for an extended period of time. CDK4/6 inhibitors have a higher response rate than the other agents. The ORR in this study was 58.4%. They are currently the first-line drugs of choice for metastatic HR+/HER2- breast cancer. Real-world data with a larger patient sample size is necessary to differentiate between the effects of the three CDK4/6 inhibitors currently available.
